# Development of diagnostic and prognostic biomarker models for knee osteoarthritis based on NLRP3 inflammasome activation

**DOI:** 10.5937/jomb0-60351

**Published:** 2026-01-28

**Authors:** Shui Xiong, Junxin Zhou, Yuying Dong, Ling Long, Gaorong Deng

**Affiliations:** 1 Affiliated Rehabilitation Hospital of Nanchang University, Department of Orthopaedic, Nanchang, China; 2 Scoliosis Center, Affiliated Rehabilitation Hospital of Nanchang University, Nanchang, China; 3 Department of Orthopaedic, Jiangxi Province Jiujiang Hospital of Traditional Chinese Medicine, Jiujiang, China

**Keywords:** NLRP3, IL-1b, IL-18, GSDMD, Knee osteoarthritis, NLRP3, IL-1 b, IL-18, GSDMD, Osteoartritis kolena

## Abstract

**Background:**

This study aimed to characterize the expression profiles of the NOD-like receptor pyrin domain-containing protein 3 (NLRP3) inflammasome and its downstream effectors [Interleukin (IL)-1b, IL-18, and Gasdermin-D (GSDMD)] in degenerative knee osteoarthritis (KOA) and to establish an integrated biomarker model for predicting the likelihood of unfavorable rehabilitation outcomes.

**Methods:**

We conducted a retrospective study involving 121 KOA patients and 94 age-matched healthy controls. Serum concentrations of NLRP3, IL-1 b, and IL-18 were quantified using ELISA, while GSDMD expression in peripheral blood mononuclear cells was assessed through flow cytometry. Conventional inflammatory markers (CRP ESR, and WBC) and neutrophil-to-lymphocyte ratio (NLR) were measured using automated analyzers. Receiver operating characteristic (ROC) curve analysis and multivariate logistic regression were performed to evaluate the diagnostic and prognostic utility of the integrated biomarker model.

**Results:**

KOA patients exhibited significantly elevated levels of NLRP3, IL-1 b, IL-18, and GSDMD compared to healthy controls (P &lt; 0.05). These biomarkers showed positive correlations with systemic inflammatory markers (CRP ESR) and negative associations with knee joint range of motion (ROM) (P &lt; 0.05). The integrated biomarker model demonstrated robust diagnostic performance for KOA (AUC = 0.928, sensitivity 84.30% , specificity 87.23%). Notably, among treated patients, those with poor recovery (n=37) maintained significantly higher post-treatment levels of NLRP3 pathway components than those with favorable recovery (P &lt; 0.05). The predictive model achieved excellent performance in identifying patients at risk of suboptimal rehabilitation (AUC = 0.911, sensitivity 94.59%, specificity 73.81%).

**Conclusions:**

Our findings highlight the pivotal role of NLRP3 inflammasome activation and GSDMD-dependent pyroptosis in mediating poor rehabilitation outcomes in KOA. The predictive model achieved excellent performance in identifying patients at risk of suboptimal rehabilitation (AUC = 0.911, sensitivity 94.59%, specificity 73.81%).

## Abstract (sr)

**Uvod:**

Cilj ove studije je bio da se okarakterišu profili ekspresije inflamazoma NOD-like receptor pirin domain containing protein 3 (NLRP3) i njegovih nizvodnih efektora [interleukin (IL)-1 b, IL-18 i gasdermin-D (GSDMD)] kod degenerativnog osteoartritisa kolena (KOA) i da se uspostavi integrisani model biomarkera za predviđanje verovatnoće nepovoljnih ishoda rehabilitacije.

**Metode:**

Sproveli smo retrospektivnu studiju koja je obuhvatila 121 pacijenta sa akutnom atrofijom kolena (KOA) i 94 zdrave kontrolne osobe odgovarajuće starosti. Serumske koncentracije NLRP3, I L-1 b i IL-18 su kvantifikovane pomoću ELISA testa, dok je ekspresija GSDMD u mononuklearnim ćelijama periferne krvi procenjena protokom citometrijom. Konvencionalni inflamatorni markeri (CRP SE i WBC) i odnos neutrofila i limfocita (NLR) su mereni pomoću automatizovanih analizatora. Analiza ROC krive (Receiver Operating Characteristic) i multivarijantna logistička regresija su izvršene kako bi se procenila dijagnostička i prognostička korisnost integrisanog modela biomarkera.

**Rezultati:**

Pacijenti sa KOA pokazali su značajno povišene nivoe NLRP3, IL-1 b, IL-18 i GSDMD u poređenju sa zdravim kontrolama (P &lt; 0,05). Ovi biomarkeri su pokazali pozitivne korelacije sa sistemskim inflamatornim markerima (CRP ESR) i negativne povezanosti sa obimom pokreta kolenskog zgloba (ROM) (P &lt; 0,05). Integrisani model biomarkera je pokazao robusne dijagnostičke performanse za KOA (AUC = 0,928, osetljivost 84,30% , specifičnost 87,23%). Primetno je da su među lečenim pacijentima, oni sa lošim oporavkom (n = 37) zadržali značajno više nivoe komponenti NLRP3 puta nakon tretmana u odnosu na one sa povoljnim oporavkom (P &lt; 0,05). Prediktivni model je postigao odlične performanse u identifikaciji pacijenata sa rizikom od suboptimalne rehabilitacije (AUC = 0,911, osetljivost 94,59%, specifičnost 73,81%).

**Zaključak:**

Naši nalazi ističu ključnu ulogu aktivacije inflamazoma NLRP3 i GSDMD-zavisne piroptoze u posredovanju loših ishoda rehabilitacije kod KOA. Prediktivni model je postigao odlične performanse u identifikaciji pacijenata sa rizikom od suboptimalne rehabilitacije (AUC = 0,911, osetljivost 94,59%, specifičnost 73,81%).

## Introduction

Knee osteoarthritis (KOA) represents one of the most prevalent chronic degenerative joint disorders that disproportionately affects middle-aged and elderly populations globally. Pathologically defined by progressive articular cartilage erosion, synovial inflammation, and aberrant bone remodeling, the disease is clinically manifested as persistent joint pain, stiffness, and progressive functional impairment, contributing to substantial reductions in quality of life while imposing significant socioeconomic burdens [1]. Current therapies (e.g., pharmacotherapy, surgery) show limited efficacy in KOA. Notably, 30-40% of patients respond poorly to rehabilitation. The molecular drivers of this heterogeneity remain unclear, hindering precision medicine [2]. Notably, heterogeneous treatment responses are frequently observed, with 30-40% of patients failing to achieve satisfactory functional recovery despite standardized rehabilitation protocols [3]. However, the molecular mechanisms driving these variations, along with clinically actionable biomarkers, remain elusive - posing a pivotal challenge that must be resolved to advance precision medicine in KOA management.

Emerging evidence has progressively illuminated the pivotal involvement of inflammatory pathways in KOA pathogenesis [4]. The NOD-like receptor pyrin domain-containing protein 3 (NLRP3) inflammasome serves as a critical regulatory nexus in the innate immune system. Upon activation, it initiates caspase-1-mediated proteolytic processing, leading to the maturation and secretion of pro-inflammatory cytokines Interleukin (IL)-1β and IL-18. This cascade perpetuates a state of chronic low-grade intra-articular inflammation and accelerates cartilage extracellular matrix degradation, mechanisms that have been definitively linked to KOA disease progression [5]. Nevertheless, while existing research has predominantly examined the NLRP3 pathway's involvement in KOA pathogenesis [6] [7], there remains a conspicuous gap in systematic investigations correlating its dynamic expression patterns with functional recovery outcomes. While conventional biomarkers (CRP ESR) reflect systemic inflammation, they lack specificity to intra-articular pathophysiology and show poor correlation with rehabilitation responses [8]. Novel biomarkers capturing joint-specific inflammatory cascades are urgently needed.

This study is the first to integrate Gasdermin-D (GSDMD)-mediated pyroptosis with NLRP3 inflammasome activation as a prognostic tool for KOA rehabilitation outcomes - including its core components (NLRP3, caspase-1) and downstream effectors (IL-1β, IL-18, GSDMD) - as molecular biomarkers for constructing a comprehensive risk prediction model of suboptimal rehabilitation in degenerative KOA. The anticipated outcomes will yield three transformative contributions: (1) elucidating the causal relationship between temporal NLRP3 inflammasome activation patterns and functional recovery trajectories; (2) establishing a novel molecular taxonomy to refine KOA subtyping; and (3) overcoming the inherent limitations of conventional assessment paradigms through advanced multi-omics integration for early risk stratification. These advances will catalyze a paradigm shift from reactive, symptom-driven management to proactive, precision rehabilitation strategies, demonstrating significant clinical translational value and interdisciplinary innovation significance.

## Materials and methods

### Study design

**Figure 1:**
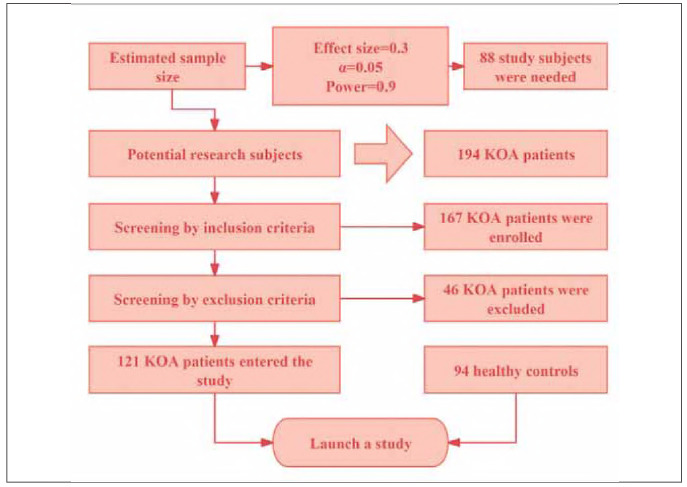
Screening process of research subjects.

This retrospective study enrolled patients diagnosed with KOA who were admitted to our hospital between March 2023 and October 2024. Data were extracted from our hospital's electronic medical record system (EMRS v3.0, Epic Systems). The participant selection process is illustrated in Figure 1, with a final cohort comprising 121 KOA patients and 94 age-matched healthy controls from the same period. The study was approved by the Institutional Ethics Committee, and written informed consent was obtained from all participants.

### Inclusion and exclusion criteria

Inclusion criteria: (1) Diagnosis of KOA according to established guidelines [9]; (2) Kellgren-Lawrence (K-L) grade [10] II-III; (3) Age ≥60 years; (4) Complete clinical records; (5) Absence of drug allergies or cognitive impairment.

Exclusion criteria: (1) Presence of joint effusion; (2) Multiple organ dysfunction; (3) Active infectious diseases; (4) Concurrent knee joint disorders; (5) Previous treatment for KOA prior to enrollment; (6) Poor compliance or inability to participate in study procedures.

Healthy controls were age/sex-matched volunteers from routine health screenings during the same period, excluding joint disorders or systemic inflammation.

### Methods

All enrolled patients underwent a standardized 12-week treatment regimen consisting of oral non-steroidal anti-inflammatory drugs (NSAIDs), intra-articular glucocorticoid injections, and supervised exercise rehabilitation. NSAID: Celecoxib 200 mg BID orally. Glucocorticoids: Triamcinolone acetonide 40 mg intra-articular, quarterly. Exercise: Supervised quadriceps strengthening (30 min/day, 5x/week). Knee joint range of motion (ROM) was assessed before and after the intervention. Additionally, patients were followed for 3 months post-treatment. Functional outcomes were evaluated using the Western Ontario and McMaster Universities Arthritis Index (WOMAC) [11] at baseline and the 3-month follow-up. Treatment efficacy was categorized as follows: WOMAC improvement ≥50% indicated favorable recovery, while<50% improvement was classified as poor recovery.

### Laboratory tests

Laboratory technicians were blinded to patient groups during biomarker assays. Fasting venous blood samples were obtained from the cubital vein of both the control group and KOA patients at baseline and post-treatment (Blood samples were collected between 7-9 am after overnight fasting). Serum was isolated via centrifugation (1500xg, 15 min), and ELISA kits (Wuhan Vector Science Co., Ltd.) were employed to quantify serum levels of NLRP3 (EF010532) along with its downstream inflammatory cytokines, IL-1β (ELA-E0563h) and IL-18 (ELI-31098h). Additionally, peripheral blood mononuclear cells (PBMCs) were isolated using Ficoll-Paque^TM^ PLUS (Cytiva, Sweden, #17144003) density gradient centrifugation at 400xg for 30 min at 20°C, which were subsequently resuspended in PBS supplemented with 1% BSA. Following fixation with 4% paraformaldehyde (15 min) and permeabilization with 0.1% Triton X-100 (10 min, 4°C), cells were incubated with an anti-human GSDMD primary antibody (1: 100 dilution, Abcam, ab219800) under light-protected conditions for 30 min. After PBS washes, a fluorescent secondary antibody (1: 200 dilution) was applied, and GSDMD-positive cell populations were analyzed using a BD FACS Canto II flow cytometer. Serum C-reactive protein (CRP) levels were assessed via an automated biochemical analyzer, while erythrocyte sedimentation rate (ESR) was determined using an automated ESR analyzer (Kate XC-A30). The white blood cell count (WBC) and the neutrophil-to-lymphocyte ratio (NLR) were measured using an automated hematology analyzer (Mindray, BC-6800).

### Statistical analysis

All statistical analyses were conducted using SPSS26.0 (IBM, USA). Categorical variables were compared using the chi-square test. Continuous variables conforming to a normal distribution were analyzed with the independent samples t-test, whereas non-normally distributed variables were evaluated using the nonparametric Mann-Whitney U test. Pre- and post-treatment comparisons were made using paired t-tests. Correlation analyses were performed using Pearson's correlation coefficients. Receiver operating characteristic (ROC) curve analysis was employed to assess diagnostic efficacy. For combined biomarker evaluation, a predictive model was first established via binary logistic regression to generate a composite formula, after which ROC analysis was applied. Missing data (<2% of variables) were handled via multiple imputation using fully conditional specification (MICE package in R). P-values from biomarker comparisons were adjusted via Holm-Bonferroni correction. Adjusted P< 0.05 was deemed significant.

## Results

### Comparability analysis between KOA patients and controls

KOA patients and healthy controls demonstrated no significant differences in demographic characteristics such as age, sex, or body mass index (BMI) (*P* > 0.05, Table 1), confirming the comparability of the two groups.

**Table 1:** No difference in baseline information between healthy controls and KOA patients.

Groups	n	Male/female	Age	BMI<br>(kg/m^2^)	Comorbidities		
					Diabetes	Hyperlipidemia	Hypertension
Healthy controls	94	62 (65.95)/32 (34.04)	66.40 ± 4.00	22.63 ± 3.51	29 (30.85)	32 (34.04)	41 (43.62)
KOA patients	121	86 (71.07)/35 (28.93)	6 7.2 5 ± 5.1 3	23.14 ± 2.64	47 (38.84)	46 (38.02)	43 (35.54)
Statisticians		χ^2^= 0.646	t = 1.3 51	t= 1.208	χ^2^= 1.479	χ^2^= 0.361	χ^2^ = 1.451
* P *		0.422	0.178	0.228	0.224	0.548	0.228

### Expression of NLRP3 and downstream effectors in KOA

**Figure 2:**
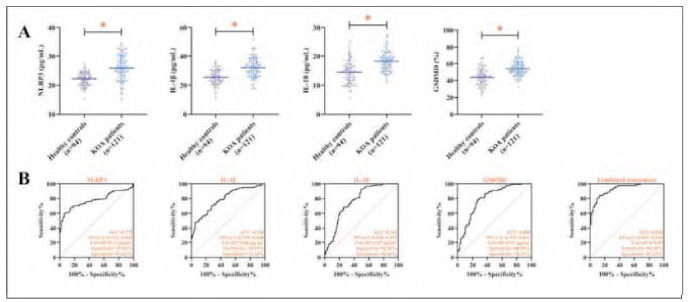
NLRP3, IL-1β, IL-18 and GSDMD are elevated in patients with KOA. (A) demonstrates the comparison of NLRP3, IL-1β, IL-18, GSDMD in KOA patients and healthy controls. (B) demonstrates the ROC curves of NLRP3, IL-1β, IL-18, and GSDMD for the diagnosis of KOA. *P< 0.05.

KOA patients displayed markedly increased serum concentrations of NLRP3, IL-1β, IL-18, and GSDMD compared to controls (*P<* 0.05, Figure 2A). Logistic regression modeling demonstrated that the NLRP3, IL-1β, IL-18 and GSDMD all had a significant effect on KOA occurrence (*P<* 0.01, Table 2). Subsequently, by ROC curve analysis, we found that the diagnostic sensitivity, specificity of the combined test of NLRP3, IL-1β, IL-18 and GSDMD for the occurrence of KOA was 84.30%, 87.23% (*P<* 0.001, AUC = 0.928, Figure 2B), which surpassed the predictive value of any single marker alone.

**Table 2:** Logistic regression analysis of the relationship between NLRP3, IL-1β, IL-18, GSDMD and KOA. Note: regression coefficient, B; standard error, S.E.; odds ratio, OR; 95% confidence interval, 95%CI.

Targets	B	S.E.	Wals	P	OR	95%CI
NLRP3	0.256	0.065	15.440	< 0.001	1.292	1.137~1.468
IL-1β	0.176	0.041	18.892	< 0.001	1.193	1.102~1.291
IL-18	0.216	0.062	11.970	0.001	1.241	1.098~1.402
GSDMD	0.136	0.029	22.533	< 0.001	1.146	1.083~1.213
Constant	-21.092	2.857	54.509	< 0.001	-	-

### Association between NLRP3/downstream effectors and KOA progression

**Figure 3:**
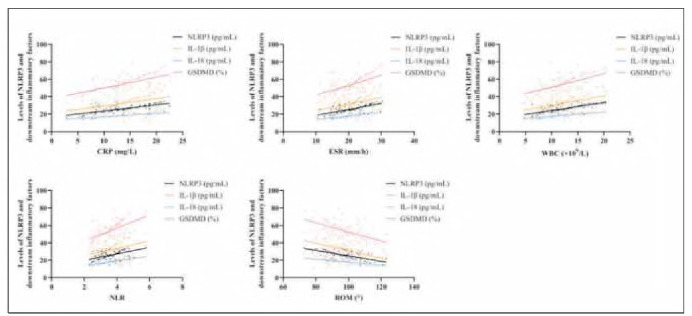
Correlation of NLRP3, IL-1β, IL-18, GSDMD and CRP ESR, WBC, NLR, ROM by Pearson's correlation coefficient. NLRP3, IL-1β, IL-18, GSDMD were positively correlated with CRP ESR, WBC, NLR and negatively correlated with ROM.

Systemic inflammatory markers in KOA patients, including CRP ESR, WBC, and NLR, were recorded as (13.35±4.34) mg/L, (20.91 ±4.23) mm/h, (12.08±3.37)x10^9^/L, and (3.61±0.63), respectively. Notably, Pearson's correlation analysis identified strong positive associations between NLRP3, IL-1β, IL-18, GSDMD, and these inflammatory indices. In contrast, NLRP3, IL-1β, IL-18, and GSDMD showed a significant inverse correlation with ROM (97.07±9.06)° (*P<* 0.05, Figure 3 and Table 3).

**Table 3:** Correlation between NLRP3, IL-1β, IL-18, GSDMD and CRP ESR, WBC, NLR, ROM (r values).

Targets	CRP	ESR	WBC	NLR	ROM
NLRP5	0.701	0.675	0.706	0.555	-0.655
IL-1β	0.540	0.475	0.546	0.589	-0.595
IL-18	0.500	0.542	0.494	0.508	-0.460
GSDMD	0.644	0.575	0.622	0.561	-0.572

### Changes in NLRP3 and downstream effectors before and after treatment

**Figure 4:**
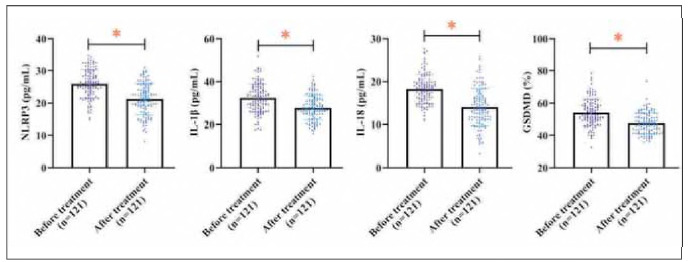
Changes in treatment of NLRP3, IL-1β, IL-18, and GSDMD in KOA patients. *P< 0.05.

Following treatment, KOA patients exhibited significant reductions in NLRP3, IL-1β, IL-18, and GSDMD levels compared to baseline measurements (*P<* 0.05, Figure 4).

### Association between NLRP3/downstream effectors and poor recovery in KOA

**Figure 5:**
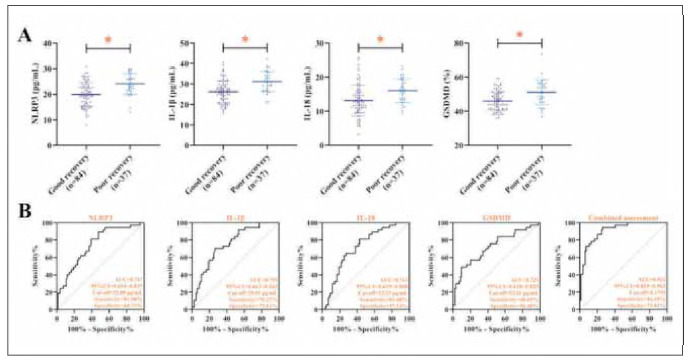
NLRP3, IL-1β, IL-18, GSDMD was higher in poorly recovered patients than in good recovered patients. (A) demonstrates the comparison of NLRP3, IL-1β, IL-18, GSDMD between well and poorly recovered patients. (B) demonstrates the ROC curve of NLRP3, IL-1β, IL-18, GSDMD for predicting poor recovery in patients with KOA. *P< 0.05.

During follow-up, 37 patients exhibited poor clinical recovery. Notably, post-treatment levels of NLRP3, IL-1β, IL-18, and GSDMD remained significantly elevated in these patients compared to those with favorable outcomes (*P<* 0.05, Figure 5A). Similarly, NLRP3, IL-1β, IL-18, GSDMD were also strongly associated with recovery from KOA by logistic regression analysis (*P<* 0.01, Table 4). Importantly, the combined evaluation of these biomarkers showed strong predictive performance for poor recovery, achieving 94.59% sensitivity and 73.81% specificity, with an AUC of 0.911 (*P<* 0.001, Figure 5B).

**Table 4:** Logistic regression analysis of the relationship between NLRP3, IL-1β, IL-18, GSDMD and recovery from KOA.

Targets	B	S.E.	Wals	P	OR	95%CI
NLRP3	0.312	0.076	17.083	< 0.001	1.366	1.178~1.584
IL-1β	0.230	0.062	13.940	< 0.001	1.258	1.115~1.419
IL-18	0.173	0.068	6.552	0.010	1.189	1.041~1.358
GSDMD	0.135	0.050	7.381	0.007	1.145	1.038~1.263
Constant	-23.345	4.561	26.197	< 0.001	-	-

## Discussion

This study conducted a systematic analysis of the expression profiles of the NLRP3 inflammasome and its downstream effectors (IL-1β, IL-18, and GSDMD) in KOA, uncovering their significant correlation with rehabilitation outcomes. The findings demonstrated markedly elevated levels of NLRP3 pathway-related factors in KOA patients, which exhibited a positive correlation with inflammatory markers (CRP ESR, and NLR) and an inverse correlation with knee joint ROM. Although post-treatment levels of these factors declined, the poor recovery group retained substantially higher residual expression compared to the favorable recovery group. Notably, the combined assessment of NLRP3, I L-1β, IL-18, and GSDMD proved effective in predicting the risk of suboptimal recovery, offering a novel approach for the precision management of KOA.

The study further validated that the NLRP3 inflammasome, a pivotal regulator of the innate immune system, drives caspase-1-dependent maturation and secretion of IL-1β and IL-18 upon activation, directly participating in KOA pathogenesis [12]. In addition, KOA patients were found to exhibit elevated serum NLRP3 levels, which may be closely associated with synovitis and cartilage degradation. Supporting evidence from an animal study by Yang C et al. [13] revealed that NLRP3-knockout mice exhibited significantly reduced cartilage degeneration and osteophyte formation, highlighting its therapeutic potential. Moreover, NLRP3 exacerbates inflammatory cascades by activating the NF- B pathway, establishing a self-amplifying feedback loop that disrupts joint homeostasis [14] and accelerates KOA progression. As central pro-inflammatory effectors downstream of NLRP3, IL-1β, and IL-18 play critical roles in sustaining the inflammatory cascade [15]. The present study revealed that both factors were markedly upregulated in KOA patients and exhibited a positive correlation with systemic inflammatory indices such as CRP and ESR. IL-1β drives cartilage catabolism via MMP-13/ADAMTS-5 upregulation [16], while IL-18 amplifies Th1 responses through IFN-γ [17]. Both sustain inflammatory cascades that impede tissue repair. Consistent with our findings, a longitudinal cohort study in KOA patients identified a significant association between elevated IL-1β levels and accelerated radiographic disease progression [18]. Notably, GSDMD—the key executioner of pyroptosis—induces plasma membrane pore formation, resulting in the uncontrolled release of pro-inflammatory mediators (e.g., HMGB1, ATP) and perpetuating localized inflammation [19]. This mechanism may underlie previously unrecognized pathways of joint injury in KOA. Our study is the first to implicate GSDMD in KOA rehabilitation outcomes, demonstrating that patients with poor recovery exhibit significantly higher GSDMD-positive PBMCs. This suggests that pyroptosis-driven release of damage-associated molecular patterns may worsen joint pathology. Supporting this notion, emerging research indicates that GSDMD inhibition mitigates cartilage deterioration and pain-related behaviors in murine osteoarthritis models [20], highlighting its therapeutic promise for KOA management.

While current evidence substantiates the association between NLRP3, its downstream effectors, and KOA pathogenesis, the translation of these findings into clinical practice remains limited by insufficient validation studies. Our investigation demonstrated that the biomarker model integrating NLRP3, IL-1β, IL-18, and GSDMD exhibited superior diagnostic efficacy for both KOA development and unfavorable rehabilitation outcomes, thereby offering a robust tool for early risk stratification and tailored therapeutic strategies. This enhanced performance likely stems from the model's comprehensive approach: whereas single-marker analysis may yield inconsistent results due to biological variability (e.g. localized treatments or comorbidities), our combined assessment provides a holistic evaluation of the KOA pathological network by simultaneously capturing the activation state of the NLRP3 pathway (NLRP3), effector cytokines (IL-1β/IL-18), and terminal pyroptotic events (GSDMD). This model could screen high-risk patients before rehabilitation, enabling targeted interventions (e.g., NLRP3 inhibitors) for poor-prognosis subgroups. Similarly, Chen J et al. [21] also established a prediction model for poor prognosis of acute myeloid leukemia based on TXNIP/NLRP3/IL1B, and achieved excellent results. Collectively, these advances hold important implications for optimizing KOA prognosis in the future.

Nevertheless, several limitations should be acknowledged: (1) The study's single-center design and relatively small sample size may limit the generalizability of the findings. Future multicenter studies with larger cohorts and extended follow-up periods are needed to validate the model's robustness. (2) GSDMD expression in synovial fluid and its relationship with serum levels remain to be elucidated, warranting further investigation. (3) The therapeutic potential of NLRP3 inhibitors (e.g., MCC950) or IL-1β-targeted agents (e.g., Anakinra) in clinical applications requires additional experimental and clinical validation.

## Conclusion

Elevated levels of NLRP3 and its downstream effectors (IL-1β, IL-18, and GSDMD) were observed in KOA patients. The multi-marker model established in this study exhibited strong predictive value for KOA progression and suboptimal rehabilitation outcomes. The model provides a molecular framework for future stratified treatment trials targeting NLRP3 pathway inhibition.

## Dodatak

### Consent to publish

All authors gave final approval of the version to be published.

### Availability of data and materials

The data that support the findings of this study are available from the corresponding author upon reasonable request.

### Funding

This work was supported by grants from Jiangxi Provincial Department of Science and Technology Incubation Project( NO.20212BAG70004).

### Acknowledgements

Not applicable.

### Conflict of interest statement

All the authors declare that they have no conflict of interest in this work.
